# Case Report: Testicular Sarcoidosis: The Diagnostic Role of Contrast-Enhanced Ultrasound and Review of the Literature

**DOI:** 10.3389/fmed.2020.610384

**Published:** 2021-01-27

**Authors:** Antonio De Cinque, Beniamino Corcioni, Martina Sofia Rossi, Alessandro Franceschelli, Fulvio Colombo, Rita Golfieri, Matteo Renzulli, Caterina Gaudiano

**Affiliations:** ^1^Department of Radiology, Azienda Ospedaliero-Universitaria di Bologna, Bologna, Italy; ^2^Department of Urology, Azienda Ospedaliero-Universitaria di Bologna, Bologna, Italy; ^3^Andrology-Unit, Azienda Ospedaliero-Universitaria di Bologna, Bologna, Italy

**Keywords:** andrology, urology, sarcoidosis, ultrasonography, contrast media

## Abstract

Sarcoidosis is a multisystemic disease histologically characterized by non-caseating epithelioid granulomas and multinucleated giant cells; the etiology is still uncertain, and likely related to a complex interplay between environmental and genetic factors. The genitourinary system is affected in fewer than 0.2% of all clinically diagnosed cases of sarcoidosis and in 5% of those identified in autopsy studies. In this report, we describe a case of a 42–year-old male with one hypoechoic lesion per testis on B-mode evaluation; contrast-enhanced ultrasound (CEUS) on both lesions was carried out. During the early phase, the masses showed a hypovascular appearance as compared to the surrounding testicular tissue, maintaining the hypo-enhancement in the late phase. Tissue biopsy for pathological evaluation confirmed testicular sarcoid involvement, showing non-caseating granulomas. Allowing visualization of testicular microvascularisation, CEUS may play an important role in excluding malignancy, avoiding unnecessary aggressive treatment for benign conditions, such as sarcoidosis. A review of the literature of reported cases since 2004 of sarcoidosis involving the testis is also included.

## Introduction

Sarcoidosis is a multisystemic disease that usually affects patients in the fifth decade of their life with a variable incidence rate depending on countries and ethnic group (1). A recent study from the Mayo Clinic reported an incidence rate of 11 per 100,000 people/year among a cohort mostly composed of white people (2) while another study from the United States estimated an incidence rate of 8.1 per 100,000 people/year in Caucasians, 17.8 in African Americans, 4.3 in Hispanics and 3.2 in Asians (3). In Europe, an incidence of 11.5 per 100,000 people/year has been reported in Sweden (4) and of 5.0 in the UK (5). The incidence rate of sarcoidosis is increasing over time in developed countries, such as Korea, together with an increase in the age of diagnosis probably due to population aging (6).

Sarcoidosis is histologically characterized by non-caseating epithelioid granulomas and multinucleated giant cells. Its etiology is still uncertain, and likely related to a complex relationship between environmental and genetic factors (7–9).

Sarcoidosis is characterized by bilateral chest hilar lymphadenopathy and/or reticulonodular pulmonary infiltrates in the vast majority of cases (>90%). However, this systemic pathology can involve any organ (10): among them the genitourinary system is involved in 5% of cases identified in autoptic studies and in fewer than 0.2% of clinically diagnosed cases. According to a review published in 2004, the male genitourinary organs most frequently involved by sarcoidosis are the epididymis (73%), the testis (47%), the spermatic cord (8%) and the prostate (3%) (11). In particular, the total number of testicular sarcoidosis accounted for 28 cases in 2004. We performed a new review of the literature, finding additional cases involving the testis associated with histologically proven diagnosis.

Testicular sarcoid presentation can vary from testicular swelling to painless or painful unilateral or bilateral masses (12, 13). Especially in cases of painless unilateral mass, the differential diagnosis is complex and difficult, ranging from testicular malignancies to infections, and could result in diagnostic errors leading to inappropriate and unnecessary treatments, such as the orchiectomy (13).

Usually the achievement of a correct diagnosis of sarcoidosis with genitourinary and in particular testicular involvement relies on the association between clinical and imaging findings (14). The most utilized imaging techniques for this issue are Ultrasound (US), Magnetic Resonance Imaging (MRI) and Positron Emission Tomography-Computed Tomography (PET-CT).

New and non-invasive imaging techniques, such as contrast-enhanced US (CEUS), have recently been refined and adopted in many guidelines (15, 16). However, to the best of our knowledge, this is the first report in the English literature to explore in detail the potentiality of CEUS in a case of histologically proven testicular sarcoidosis.

A very rare case of testicular sarcoidosis is herein reported together with a detailed review of the literature highlighting the role of CEUS to confidently achieve the final diagnosis excluding other possible differential diagnoses, such as testicular tumor masses.

## Case Report

A 42–year-old Caucasian male was admitted to our Hospital referring a 4-month history of gradually increasing upper left quadrant pain. His medical history included bladder neck sclerosis, cholecystectomy, diabetes insipidus, hypogonadotropic hypogonadism, allergic asthma, and a smoking history until 10 months before this admission. The medical examination did not show any relevant features and, therefore, the subsequent diagnostic work-up continued with abdominal US.

**Figure 1:**
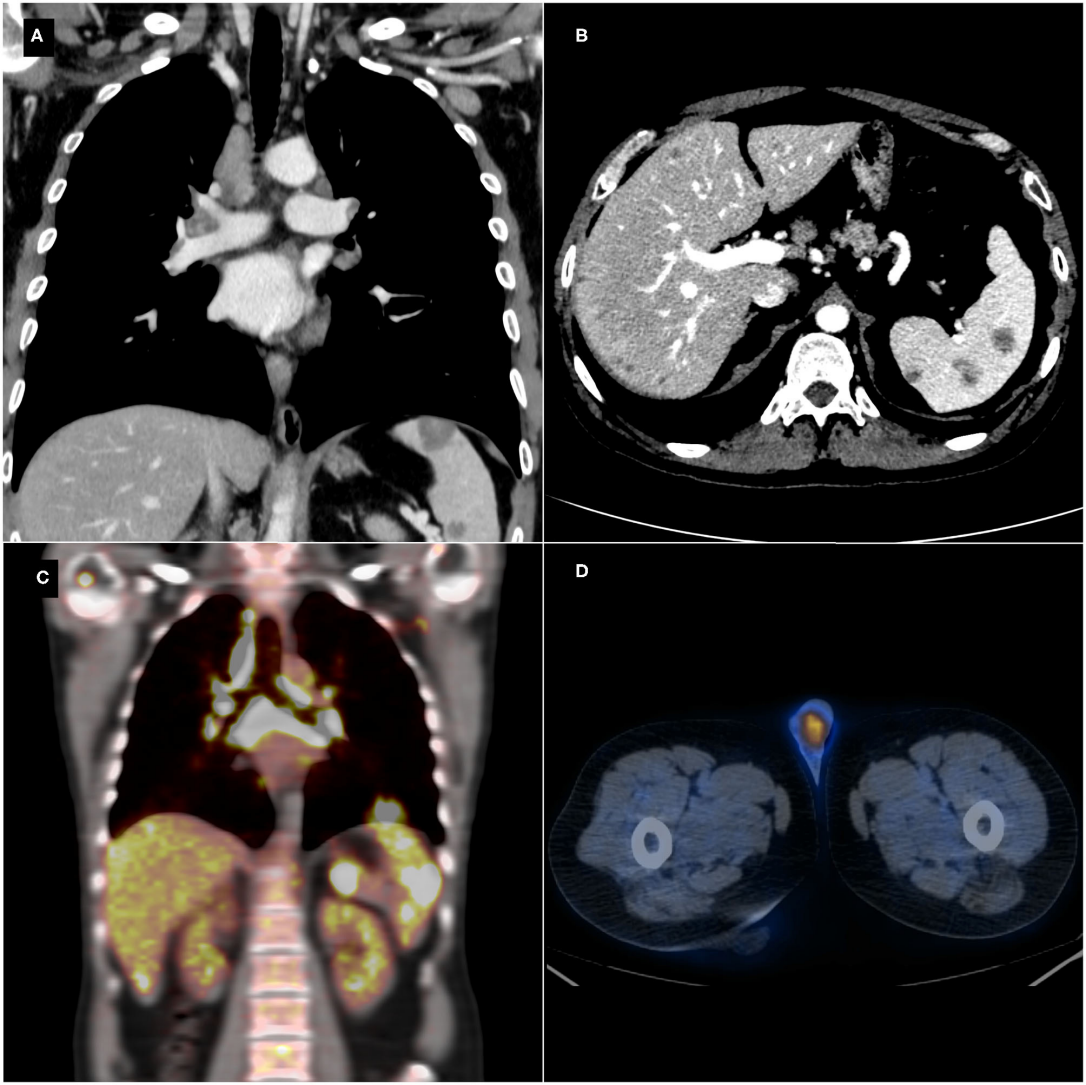
Thoraco-abdominal contrast-enhanced computed tomography (CT) scan showing hilar and mediastinal lymphadenopathies **(A)**, enlarged retroperitoneal lymph nodes, and multiple hepatic and splenic hypodense lesions **(B)**. Positron emission tomography-computed tomography (PET/CT) detecting an increased focal ^18^F-Fludeoxyglucose (^18^F-FDG) uptake in the mediastinal and abdominal lesions **(C)** and in the left testis **(D)**.

Abdominal US showed multiple hypoechoic splenic and hepatic lesions ranging from 5 to 27 mm and, therefore, the patient underwent total body CT scan for a correct lesions characterization and staging. CT scan demonstrated bilateral hilar lymphadenopathies in the chest, pulmonary perilymphatic micronodules, enlarged retroperitoneal lymph nodes, and confirmed the hepatic and splenic lesions (Figures 1A,B). The first diagnostic hypothesis was the lymphoma and a ^18^F-Fludeoxyglucose (^18^F-FDG) PET/CT was performed, showing increased ^18^F-FDG uptake (standardized uptake value (SUV) max=17 at the chest hilar lymphadenopathies level) in all the lesions revealed on CT (Figure 1C). Moreover, a focal uptake was detected in left testis (Figure 1D). A brain MRI excluded central nervous system involvement. Lymphopenia was the only abnormal blood cell count value. Alpha-fetoprotein (AFP), Human chorionic gonadotropin (HCG), Aspartate aminotransferase (AST), Alkaline phosphatase (ALP) were within their normal ranges, while alanine aminotransferase (ALT) (55 U/L), gamma-glutamyl transferase (GGT) (60 U/L), C-reactive protein (CRP) (0.82 mg/dL) and Erythrocyte sedimentation rate (ESR) (20 mm) were mildly elevated. Moreover, Angiotensin Converting Enzyme (ACE) levels were elevated (95 U/L). He had no history or signs of tuberculosis (QuantiFERON®-TB Gold Plus test was negative).

**Figure 2:**
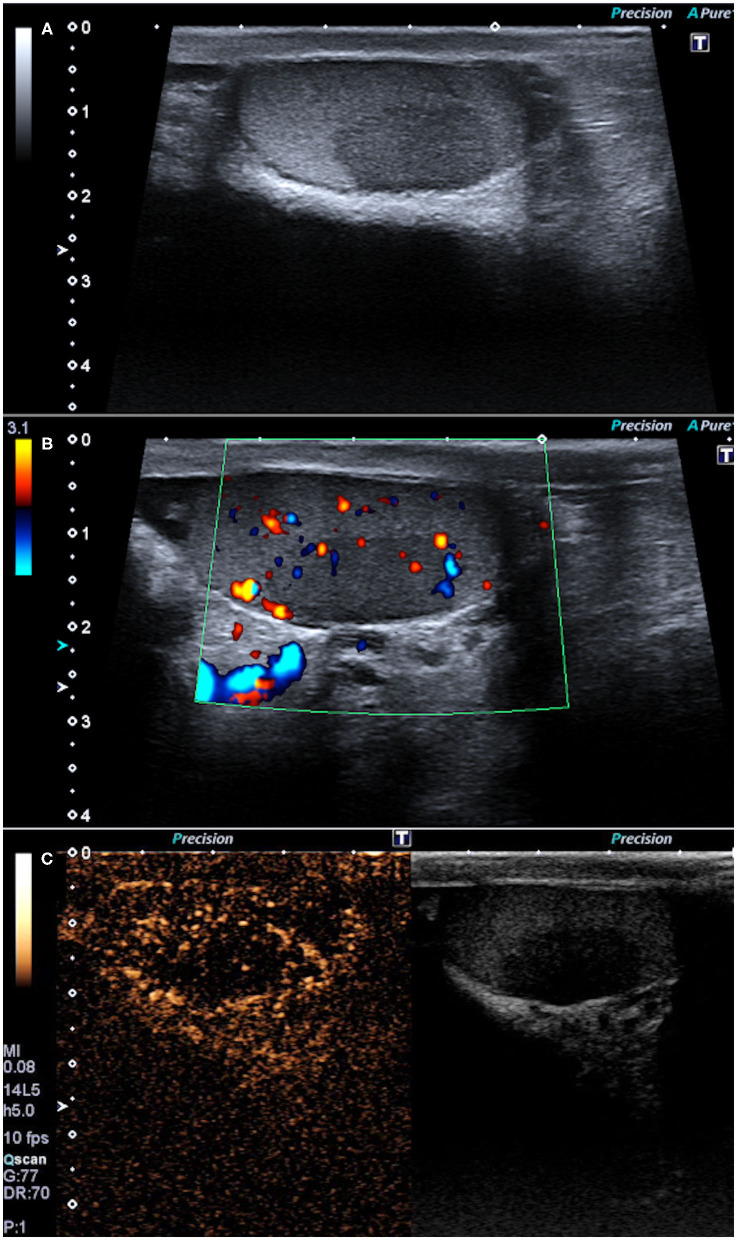
B-mode ultrasonography showing one hypoechoic lesion in the left testis with ill-defined margins **(A)** and some Color-Doppler flow **(B)**. Contrast-enhanced ultrasonography showing the hypovascular appearance of the lesions as compared to the surrounding testicular tissue **(C)**.

These findings raised the suspicion of lymphoma or sarcoidosis. An endobronchial ultrasound-guided transbronchial needle aspiration (EBUS-TBNA) was performed on the chest hilar lymphadenopathies, showing non-caseating granulomas, consistent with sarcoidosis. Stains for acid-fast bacilli and polymerase chain reaction (PCR) in specimens were negative. The final diagnosis was sarcoidosis with multiorgan involvement. A testicular US evaluation was carried out using a Canon-Toshiba Aplio 500^TM^ (Otawara, Kanto, Japan) with a high frequency (4–14 MHz) linear transducer. The US demonstrated, using the B-mode evaluation, a hypoechoic lesion of 20 mm with ill-defined margins in the left testis (Figure 2A) corresponding to the lesion identified on PET-CT; moreover, differently from the latter technique, the US identified a smaller and well-shaped hypoechoic lesion also in the right testis (6 mm). The Color Doppler demonstrated the presence of vascular flow within the lesions (Figure 2B). Therefore, it was decided to perform CEUS, conducted with the administration of 4.8 ml of second-generation contrast media (SonoVue™, Bracco, Milano, Italy) followed by 10 mL of 0.9% saline solution. Both the testicular lesions demonstrated the same pattern on CEUS. In particular, during the arterial phase, the masses showed a hypovascular appearance as compared to the surrounding testicular tissue (Figure 2C), maintaining the hypo-enhancement in the late phase. This CEUS pattern was not typical of the most frequent testicular tumors such as seminomas, which usually are arterialized appearing hyperechoic on CEUS (17). Finally, the imaging diagnosis was testicular sarcoidosis, based principally on bilateral involvement of testes on CEUS and on the CEUS pattern (hypoenhancement). Testicular sarcoid involvement was confirmed by surgical biopsy of both testicular masses that demonstrated non-caseating granulomas.

The patient was treated with corticosteroids and then with a second-line therapy (methotrexate), thus achieving a reduction of the SUVmax in all of the sarcoid lesions (SUVmax = 2.6 at the testicular level) at 6- and 12-month PET/CT follow-up.

According to our review (Table 1), from 2004 (11) until now we have identified 20 cases (including the present one) of testicular sarcoidosis. Finally, to date, the total number of histologically proven testicular sarcoidosis published in literature account for 48 cases.

**Table 1:** Histologically proven cases of sarcoidosis involving the testis reported in the English literature from 2004 to 2020 (including the present case).

**References**	**Imaging**	**Uni- or Bilateral**	**Orchiectomy**
Rees et al. (18)	US	Bilateral	No
Rehman et al. (19)	US	Bilateral	No
Massarweh et al. (12)	US	Bilateral	No
Thuret et al. (20)	US	Unilateral (R)	Yes
Real et al. (21)	US	Bilateral	Yes
Gupta and Senadhi (13)	US	Unilateral (R)	Yes
Kim et al. (22)	US	Unilateral (R)	No
Paknejad et al. (23)	US	Bilateral	No
Kovac et al. (24)	N/A	N/A	No
Esnakula et al. (25)	US	Unilateral (R)	Yes
Joel et al. (26)	US	Unilateral (L)	Yes
Patel et al. (27)	US	Bilateral	No
Knox et al. (28)	N/A	Bilateral	Yes
Chierigo et al. (29)	US	Bilateral	No
Babst et al. (30)	US	Bilateral	No
Konishi et al. (31)	US, CT	Bilateral	No
Hamitouche et al. (32)	US	Bilateral	No
Kimura et al. (33)	US, MRI, Gallium-67 scintigraphy	Bilateral	No
Parida et al. (34)	CT, FDG-PET/CT	Unilateral (L)	Yes
This study	FDG-PET/CT, US, CEUS	Bilateral	No

*US, Ultrasound; CT, Computed Tomography; MRI, Magnetic Resonance Imaging; FDG-PET/CT, Fludeoxyglucose-Positron Emission Tomography/Computed Tomography; CEUS, Contrast-enhanced ultrasound; R, right; L, left; N/A, not available*.

## Discussion

Testicular masses may have many differential diagnoses including malignancies and, very rarely, benign processes such as traumatic or infective/inflammatory lesions including sarcoidosis which incidence is increasing (6, 35). US has a pivotal role in investigating testicular lesions since it is extremely accurate in the detection of the masses, even very small ones, due to its high spatial resolution (35). Moreover, the US often represents the sole imaging technique needed prior to surgery, although the recognition of benign entities may be challenging (36). The most common US findings in testicular sarcoidosis are multifocal small hypoechoic lesions which can range from a few millimeters to a few centimeters, with well-circumscribed or ill-defined margins. Bilateral unifocal lesions, as in the present case, are less common (37).

However, a question remains unsolved: what is the correct diagnosis of the left testicular lesion? In fact, the testicular sarcoid involvement is very rare, and an association between testicular cancer and sarcoidosis has been reported (38). Moreover, the ^18^F-FDG uptake was detected only in the left testis (not in both testes) and the max SUV of the left testicular lesion (SUVmax = 11.6) was different from the sarcoid lesions involving the other organs. All these findings could not allow the exclusion of a testicular tumor.

In our case, on Color Doppler US some areas of vascularity within the lesion were detected. Usually, the color flow detectable in primary tumors of the testis using this imaging technique is higher than that of non-neoplastic lesions. However, some malignant lesions of the testis could lack internal vascularity on Color Doppler, such as burned-out testis tumors, being difficult to characterize with respect to non-neoplastic lesions (39). Therefore, the sole use of Color-Doppler US was not sufficient to exclude the possible diagnosis of testicular tumor lesion. Furthermore, ^18^F-FDG-PET scan has high sensitivity in detecting lesions having an increased glucose uptake, but it is unable to differentiate an inflammatory process such as sarcoidosis from malignancy (40). Furthermore, in our case, the right testicular lesion did not demonstrate ^18^F-FDG uptake probably due the small lesion dimension under the resolution power of this technique.

In the present case, the testicular unifocal lesions did not demonstrate hyperenhancement on CEUS and, moreover, the lesions were bilateral. The CEUS hyperenhancement of a testicular lesion has a positive predictive value of 97% for neoplasia and, although its presence alone is not specific enough to establish an unequivocal diagnosis, it is suggestive for a neoplastic testicular lesion, including malignancy (41). Moreover, the most common testicular malignancies in the same age of the patient described in this case are the testicular germ cell tumors that are bilateral in only 2% of cases (42).

The sarcoid testicular masses could pose problems of differential diagnosis with other bilateral testicular lesions that appear hypoechoic on B-mode US (Table 2), such as Seminoma, Leydigiomas, lymphomas, and testicular adrenal rest tumors. Seminomas usually demonstrate rapid wash-in coupled with rapid washout on CEUS (17). Leydigiomas show hyperenhancement as compared to the surrounding testicular parenchyma on CEUS, with delayed wash-out (17) and therefore the differential diagnosis with sarcoid testicular masses appear easy to perform. Lymphomas, along with leukemia, show marked CEUS hypervascularization (visible also with Color Doppler), a “nonbranching linear pattern” (known also as “straight vessel pattern”) and a rapid filling time (43, 44), differently from sarcoid testicular masses. Testicular adrenal rest tumors demonstrate high hypervascularization as compared to the sarcoid lesions; moreover, these rare lesions arise in younger patients with congenital adrenal hyperplasia (45). Different approach could be have in case of oncological patients, in whom metastases (the most common are from prostate carcinoma, melanoma, colon and kidney cancer) are rare but could not be excluded especially in cases of advanced malignancy (46).

**Table 2:** Differential diagnosis of testicular sarcoidosis with the most frequent testicular lesions on B-Mode ultrasound and contrast-enhanced ultrasound.

	**B-Mode US**	**CEUS**
Seminoma	Hypoechoic	Fast wash-in and rapid washout
Leydigioma	Hypoechoic	Wash-in and delayed washout
Sarcoidosis	Hypoechoic	Hypo-enhancement
Lymphoma	Hypoechoic	Fast wash-in and rapid washout (“straight vessel pattern”)^*^
Testicular adrenal rest tumors	Hypoechoic	Fast wash-in and delayed washout

* *“Straight vessel pattern” (or “nonbranching linear pattern”) of increased vascularity represents a parallel arrangement of testicular small vessels that reflects the interstitial growth pattern of lymphoma, which preserves the vascular architecture of the testis*.

*US, Ultrasound; CEUS, Contrast-enhanced ultrasound*.

A possible limitation of our case was the absence of MRI evaluation. However, MRI is not able to identify a specific pattern for sarcoidosis (47), and therefore it was not performed after CEUS in our case.

In conclusion, CEUS could play an important role in the evaluation of testicular lesions, and in particular of benign lesions, such as sarcoidosis. Due to the rising incidence rate of this disease and the reported association with testicular cancer, the need to establish a correct differential diagnosis will likely increase over time. CEUS could allow to achieve a correct diagnosis of testicular lesions, due to its ability in identifying very small testis lesions, such as sarcoidosis, coupled with its high accuracy in excluding malignancies, thus avoiding aggressive and potentially unnecessary maneuvers, such as biopsy for benign conditions.

## Data Availability Statement

The original contributions presented in the study are included in the article/supplementary material, further inquiries can be directed to the corresponding author/s.

## Ethics Statement

Ethical review and approval was not required for the study on human participants in accordance with the local legislation and institutional requirements. The patient has given his written informed consent to publish the case (including publication of images).

## Author Contributions

All authors listed have made a substantial, direct and intellectual contribution to the work, and approved it for publication.

## Conflict of Interest

The authors declare that the research was conducted in the absence of any commercial or financial relationships that could be construed as a potential conflict of interest.
